# Recognition of Glycometabolism-Associated lncRNAs as Prognosis Markers for Bladder Cancer by an Innovative Prediction Model

**DOI:** 10.3389/fgene.2022.918705

**Published:** 2022-07-19

**Authors:** Dongdong Tang, Yangyang Li, Ying Tang, Haoxiang Zheng, Weihan Luo, Yuqing Li, Yingrui Li, Zhiping Wang, Song Wu

**Affiliations:** ^1^ Department of Urology, Lanzhou University Second Hospital, Lanzhou, China; ^2^ Institute of Urology, The Third Affiliated Hospital of Shenzhen University (Luohu Hospital Group), Shenzhen University, Shenzhen, China; ^3^ Luohu Clinical Medicine School, Shantou University Medical College, Shantou University, Shantou, China; ^4^ South China Hospital, Health Science Center, Shenzhen University, Shenzhen, China

**Keywords:** lncRNA, bladder cancer, bioinformatics, glycometabolism, prognosis prediction

## Abstract

The alteration of glycometabolism is a characteristic of cancer cells. Long non-coding RNAs (lncRNAs) have been documented to occupy a considerable position in glycometabolism regulation. This research aims to construct an effective prediction model for the prognosis of bladder cancer (BC) based on glycometabolism-associated lncRNAs (glyco-lncRNAs). Pearson correlation analysis was applied to get glyco-lncRNAs, and then, univariate cox regression analysis was employed to further filtrate survival time-associated glyco-lncRNAs. Multivariate cox regression analysis was utilized to construct the prediction model to divide bladder cancer (BC) patients into high- and low-risk groups. The overall survival (OS) rates of these two groups were analyzed using the Kaplan–Meier method. Next, gene set enrichment analysis and Cibersortx were used to explore the enrichment and the difference in immune cell infiltration, respectively. pRRophetic algorithm was applied to explore the relation between chemotherapy sensitivity and the prediction model. Furthermore, reverse transcriptase quantitative polymerase chain reaction was adopted to detect the lncRNAs constituting the prediction signature in tissues and urine exosomal samples of BC patients. A powerful model including 6 glyco-lncRNAs was proposed, capable of suggesting a risk score for each BC patient to predict prognosis. Patients with high-risk scores demonstrated a shorter survival time both in the training cohort and testing cohort, and the risk score could predict the prognosis without depending on the traditional clinical traits. The area under the receiver operating characteristic curve of the risk score was higher than that of other clinical traits (0.755 > 0.640, 0.485, 0.644, or 0.568). The high- and low-risk groups demonstrated very distinct immune cells infiltration conditions and gene set enriched terms. Besides, the high-risk group was more sensitive to cisplatin, docetaxel, and sunitinib. The expression of lncRNA AL354919.2 featured with an increase in low-grade patients and a decrease in T3-4 and Stage III–IV patients. Based on the experiment results, lncRNA AL355353.1, AC011468.1, and AL354919.2 were significantly upregulated in tumor tissues. This research furnishes a novel reference for predicting the prognosis of BC patients, assisting clinicians with help in the choice of treatment.

## Introduction

Bladder cancer (BC), causing about 13,050 deaths and accounting for nearly 4% of the total deaths in 2020, has become one of the most common malignant carcinomas over the world (Siegel et al., 2020). Pathologically, 90% of tumors originate from bladder urothelium, which is called bladder urothelial carcinomas (BLCA). Clinically, 75% of primary BC are diagnosed as non-muscular invasive (NMIBC) and the other 25% are usually muscular invasive (MIBC). The 5-year relapse-free survival rate is 43% in low-risk NMIBC patients, and it sharply decreases to 33% in medium-risk NMIBC patients. As to high-risk NMIBC patients, about 21% of them will progress to MIBC, unfortunately (Lenis et al., 2020; Ritch et al., 2020). In general, radical cystectomy combined with chemotherapy is necessary to be employed to treat MIBC, resulting in a severe reduction in life quality and prognosis of MIBC patients. As we can see, traditional prognostic evaluation indicators for BC do not work well in clinical practice. Therefore, it is imperative to develop more effective methods to assess the prognosis of BC patients.

The unique glycometabolism of tumor cells, which is quite different from healthy cells, facilitates the genesis and development of tumor. For example, abundant lactic acid can be produced in tumor cells, even though sufficient oxygen is found by the glycolytic pathway, which is known as Warburg Effect (Hanahan and Weinberg, 2011). Glycolysis can improve the tolerance of tumor cells to hypoxia and ischemia, in addition to also avoiding apoptosis caused by the inhibition of oxidative phosphorylation. In addition, the acidic tumor microenvironment resulting from the high concentration of lactic acid is able to destroy extracellular matrix, facilitating tumor infiltration and metastasis. It has been documented that the targeted regulation of glycometabolism in BC cells effectively restrains their proliferation, indicating the great significance of glycometabolism in the development of BC (Massari et al., 2016). Several key proteins involved in glycometabolism, such as glucose 6 phosphate dehydrogenase (G6PD), glucose transporter-1 (GLUT-1), demonstrate a higher expression level in BC cells, and their expressions have been reported to positively correlate with the tumor stage (Li et al., 2017; Chen et al., 2018). However, scarce exploration has been carried out to analyze the correlation between the whole glucometabolic state and the prognosis of BC patients.

LncRNAs refer to a kind of transcripts with the length more than 200 nucleotides, performing diversified tasks in cell life cycle, including nuclear domain organization, cis or trans transcriptional regulation, and RNA or protein molecule regulation (Kopp and Mendell, 2018). In the aspect of tumor regulation, lncRNAs have been recognized to involve in tumor cell growth, differentiation, apoptosis, metastasis, etc. in the last decade (Hung et al., 2011; Arase et al., 2014). Among the complicated bio-regulations, we noticed that some of lncRNAs are capable of participating in the regulation of glycometabolism in cancer cells. LncRNA expression profile in cancer cells shows a particular feature when compared with that in normal cells and demonstrates the potential to predict the prognosis of cancer patients. Whether undiscovered prognosis relations exist between glyco-lncRNAs and BC patients is of great significance to explore.

In this study, a prediction prognosis model of BC was innovatively presented based on glyco-lncRNAs with a satisfactory prediction efficacy when tested in TCGA cohort and GSE154261 cohort. The value of this signature for evaluating prognosis, immune cells infiltration, drug sensitivity was also analyzed. Gene set enrichment analysis (GSEA) was used to explore the underlining mechanisms. These 6 glyco-lncRNAs consisting of the prediction signature were detected in tissues and urine exosome samples of BC patients.

## Materials and Methods

### Patients and Clinical Samples Acquisition

The transcriptome profiling files (FPKM) and clinical data of BC patients were extracted from the Cancer Genome Atlas Genomic Data Commons (TCGA GDC). The data of GSE154261 including FPKM data and prognosis information of BC patients were downloaded from Gene Expression Omnibus (GEO). Patients diagnosed with BC, with complete clinical information and gene expression information, were enrolled in our study. Patients with a follow-up time less than 1 month were excluded from our study. After that, 411 samples were collected to acquire the glyco-lncRNAs, and 393 samples were collected to construct the prognosis prediction model in TCGA dataset. 73 samples in GSE154261 dataset were included in our study. The gene expression profiles were normalized using the scale method provided in the “limma” R package. GRCh38.p13 from Ensemble human genome browser (http://asia.ensembl.org/index.html) was used to categorize the lncRNAs and protein-coding genes. This research excluded the patients with incomplete data or ambiguous living conditions.

The BC tissues and paired normal paracancerous tissues were collected from Shenzhen Luohu People’s Hospital. The 4 urine samples of BC patients were also collected from Shenzhen Luohu People’s Hospital. A glycometabolism gene set containing 200 genes was downloaded from GSEA (https://www.gsea-msigdb.org/gsea/index.jsp). The understanding and written consent of all subject has been taken in this research. The methodologies in this study conformed to the standards set by the Declaration of Helsinki and were approved by the Ethics Committee of Shenzhen Luohu People’s Hospital.

### Construction and Verification of the Accuracy of Prognostic Model

FPKM data of 411 BC patients were divided into mRNA expression data and lncRNA expression data. According to the filter condition of |R| > 0.4 and *p* < 0.001, the expression profile about 200 glycometabolism-related genes from the mRNA expression data was further extracted, and their co-expressed lncRNAs were acquired subsequently. Combined with clinical data, these co-expressed lncRNAs were subjected to univariate cox regression analysis in order to discover the lncRNAs that were significantly related to the survival time of BC patients. To construct a prognosis model by using lycol-lncRNAs, multivariate cox regression analysis and Akaike information criteria (AIC) were selected. To conclude, 6 of glyco-lncRNAs with the best AIC value were chosen to build the prognostic model. Patients were separated into high- and low-risk groups according to the median risk value of all patients that was calculated by using the above prognostic model. Then, we compared the OS of the high- and low-risk groups and plotted patients’ risk curves. The accuracy of this prognostic model was validated in GSE154261 with the same method.

### Correlation Analysis With Clinical Characters

Multivariate COX regression and univariate COX regression analysis were used to evaluate whether the risk value, compared with other clinical characteristics, such as age, sex, Union for International Cancer Control (UICC) stage, and T stage, could predict a patient’s prognosis all alone or not. In addition, the 1-, 3-, and 5-years receiver operating characteristic (ROC) curves were plotted to assess the veracity of the prediction model we constructed. We explored the relation between the clinical traits and the 6 glyco-lncRNAs included in the prognostic model by comparing the mean expression of each lncRNA.

### Exploration of Immune Cell Infiltration, Prediction of Drug Sensitivity and Gene Set Enrichment Analysis

By consulting the website of Cibersortx (https://cibersortx.stanford.edu/), we got the immune cell infiltration condition of all the included samples. Then, we used the Wilcoxon test to compare the immune cell infiltration condition between the high-risk group and low-risk group. In order to explore the function of this prognostic model in predicting drug sensitivity, pRRophetic algorithm was used to calculate the half-maximal inhibitory concentration (IC50) of familiar drugs used for BC treatment. Wilcoxon signed-rank test was adopted to contrast the IC50 scores between the high-risk group and the low-risk group. GSEA 4.0.1 was downloaded from (https://www.gsea-msigdb.org) to explore the difference of gene set enrichment between the high- and low-risk groups. Also, FDR < 0.25 and *p* < 0.05 were set as the thresholds to obtain positive results.

### Isolation of Urine Exosomes From Bladder Cancer Patients

Exosomes were extracted by the differential centrifugation method. In detail, urine samples were centrifuged at 300 g for 10 min, 2,000 g for 10 min, and 10,000 g for 70 min at 4°C in sequence to remove the cells, cell debris, and large vesicles. The supernatant was gathered and then centrifuged at 100,000 g for 70 min at 4°C to precipitate exosomes. The pellets containing exosomes were collected and resuspended in filtered phosphate-buffered saline (PBS), and another centrifugation at 100,000 g for 70 min was carried out to eliminate protein contamination. All the centrifuge steps were kept at 4°C. The pellet enriched exosomes were resuspended in filtered PBS and stored at −80°C until use.

### Western Blotting

Urine exosomal samples were lysed with RIPA buffer containing 1% proteinase inhibitor to obtain the total proteins. The total proteins were separated by SDS-PAGE gel (12%) and passed onto PVDF (Millipore, United States) membranes. After being blocked in 5% non-fat milk for 1 h, the PVDF membranes were incubated with the exosome marker antibodies including anti-CD9, anti-CD63, and anti-CD81 (SBI, United States) overnight at 4°C. The membranes hatched with secondary antibody for 1 h at room temperature were visualized with the enhanced chemiluminescence reagent (Millipore, United States) under Model Spark.

### RNA Extraction From Tissues and Urine Exosomes

Tissue RNA samples used in this study were extracted from 10–20 mg of normal or cancer tissues by using Cell/Tissue Total RNA Kit (YEASEN Biotech, Shanghai). Exosomal RNA samples were extracted by using Trizol reagent as the manufacturer’s instruction. Next, the concentrations of RNA were measured by NanoDrop 8000 and stored at −80°C for further research.

### Reverse Transcriptase Quantitative Polymerase Chain Reaction (RT-qPCR)

Based on the sequences of lncRNAs downloaded from http://asia.ensembl.org/index.html, the primers of the involved lncRNAs for qPCR were designed by ourselves and produced by Guangzhou RuiBio BioTech. 0.5 µg of total RNA was applied for generating complementary DNA (cDNA) with Hifair™ II 1st Strand cDNA Synthesis Super Mix for qPCR (YEASEN Biotech, Shanghai). Hieff UNICON^®^ Universal Blue qPCR SYBR Green Master Mix (YEASEN Biotech, Shanghai) was used for qPCR executing by ABI 7500 Real-Time PCR system (Applied Biosystems). The expression of GAPDH was used as the internal reference. Also, the expression quantities of lncRNAs were calculated with the method of 2^−ΔΔCt^. All the primer sequences were shown in Supplementary Table S1.

### Statistical Analysis and Software Support

PERL programming langue (https://www.perl.org/), version (strawberry-perl-5.32.0.1-64bit.msi) was used to process the data. R software (https://www.r-project.org/), version R x64 4.0.2, or GraphPad Prism 8.0 (GraphPad Software, Inc) was used for statistical analysis and figure output. The standard two-tailed student t-test was used to assess the group statistical analysis. Kaplan–Meier curve with a log-rank test was adopted to assess the overall survival (OS) between different groups. *p* values less than 0.05 were considered as statistically significant (*p < 0.05, **p < 0.01).

## Results

### Glyco-lncRNAs Were Involved in the Construction of Prediction Model

**FIGURE 1:**
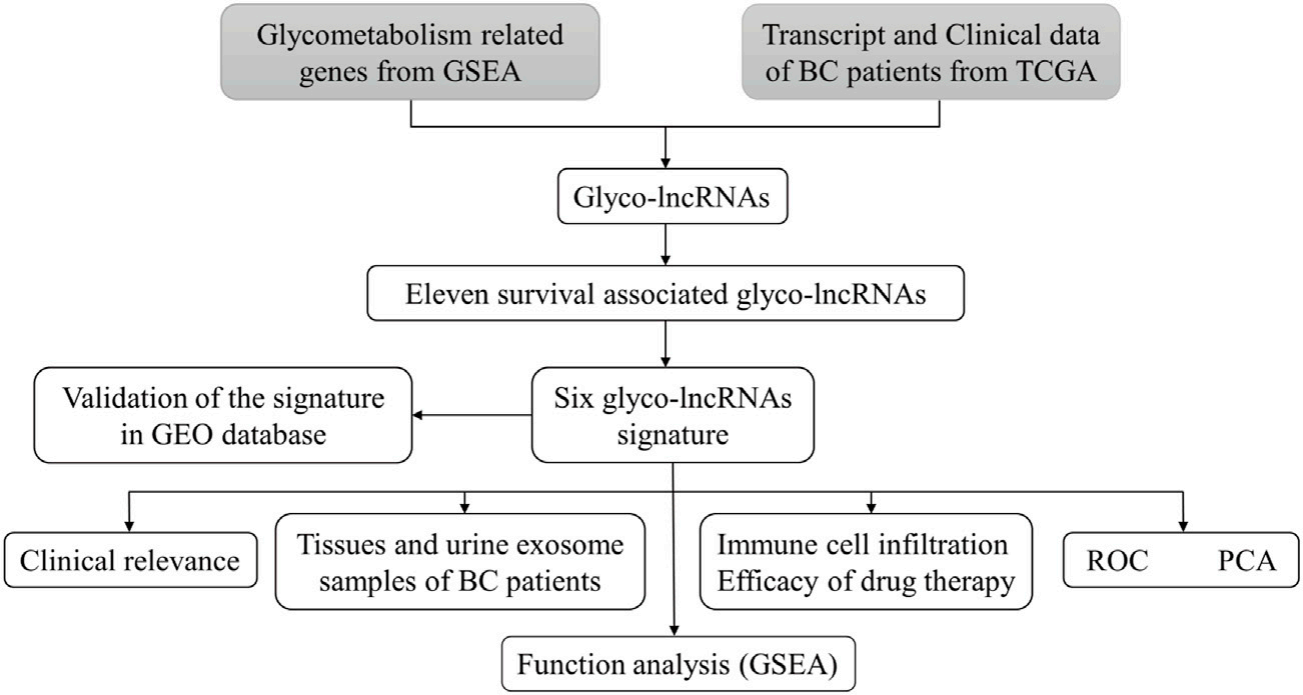
The flowchart of this study. TCGA, the cancer genome atlas; BC, bladder cancer; lncRNA, long non-coding RNA; glyco-lncRNAs, glycometabolism associated lncRNAs; GEO, the gene expression omnibus; ROC, receiver operating characteristic; PCA, principal component analysis; GSEA, gene enrichment analysis.

**FIGURE 2:**
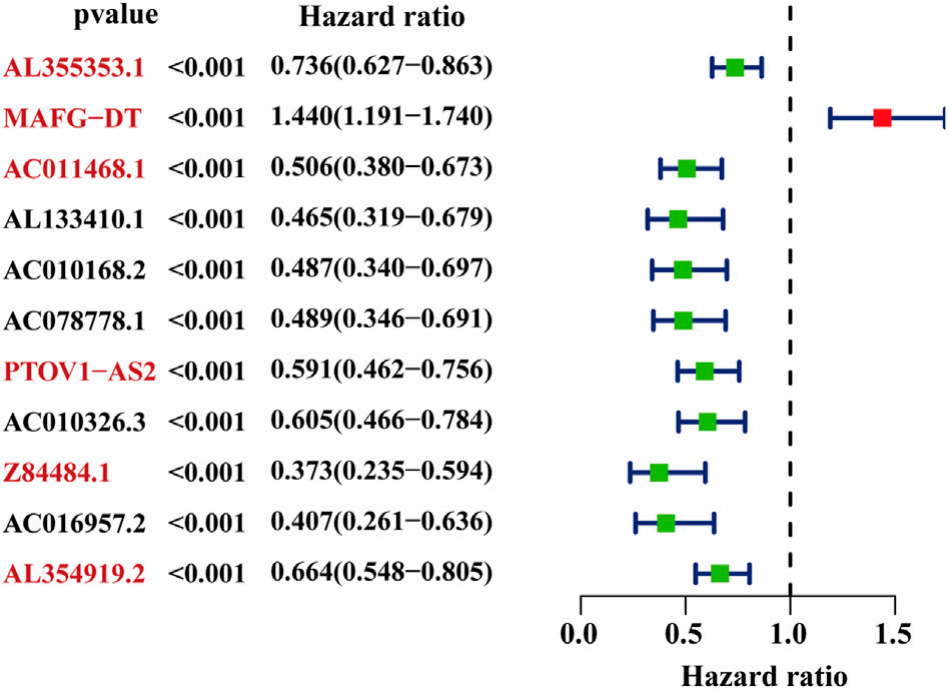
The selected glyco-lncRNAs related to the prognosis of BC patients. The lncRNAs with red front were involved in construct the prediction model.

The whole workflow of the present research was illustrated in detail in Figure 1. The result of Pearson correlation analysis manifested there were 794 lncRNAs in total associated with glycometabolism. Among them, only 11 lncRNAs were found to demonstrate a pronounced relation to the prognosis of BC patients by taking advantage of univariate cox regression analysis (Figure 2). Based on multivariate cox regression analysis, we utilized AIC to further screen these 11 lncRNAs, resulting in 6 lncRNAs with the best AIC value to construct the ideal model, the detailed information of which were shown in Table 1. According to the efficacy for OS predicted by these 6 lncRNAs (Figure 2), a risk value of the 6-lncRNA trait was calculated by the following algorithm: Risk score = (−0.174 × Expression_AL355353.1_) + (0.358 × Expression_MAFG-DT_) + (−0.435 × Expression_AC011468.1_) + (−0.290 × Expression_PTOV1-AS2_) + (−0.719 × Expression_Z84484.1_) + (−0.271 × Expression_AL354919.2_).

**TABLE 1:** Detail information of the 6 glyco-lncRNAs.

			Multivariate cox regression analysis			
Gene symbol	Ensembel ID	Description	Coefficient	HR	95%CI	*p*-value
AL355353.1	ENSG00000270761.1	CD2AP-DT-201	−0.174	0.840	0.699–1.009	0.061
MAFG-DT	ENSG00000265688.2	MAFG-DT-201	0.357	1.430	1.169–1.749	<0.001
AC011468.1	ENSG00000260160.1	—	−0.435	0.647	0.466–0.897	0.009
Z84484.1	ENSG00000224666.3	ETV7 and PTX1 antisense RNA1	−0.719	0.487	0.312–0.759	0.001
PTOV1-AS2	ENSG00000269352.1	PTOV1 antisense RNA2	−0.289	0.748	0.565–0.991	0.043
AL354919.2	ENSG00000254545.1	—	−0.270	0.762	0.622–0.935	0.009

HR, hazard ratio; CI, confidence interval.

### The Proposed Model Can Predict Prognosis of Bladder Urothelial Carcinomas Cohorts Both in the Cancer Genome Atlas Database and GSE154261 With High-Efficiency

**FIGURE 3:**
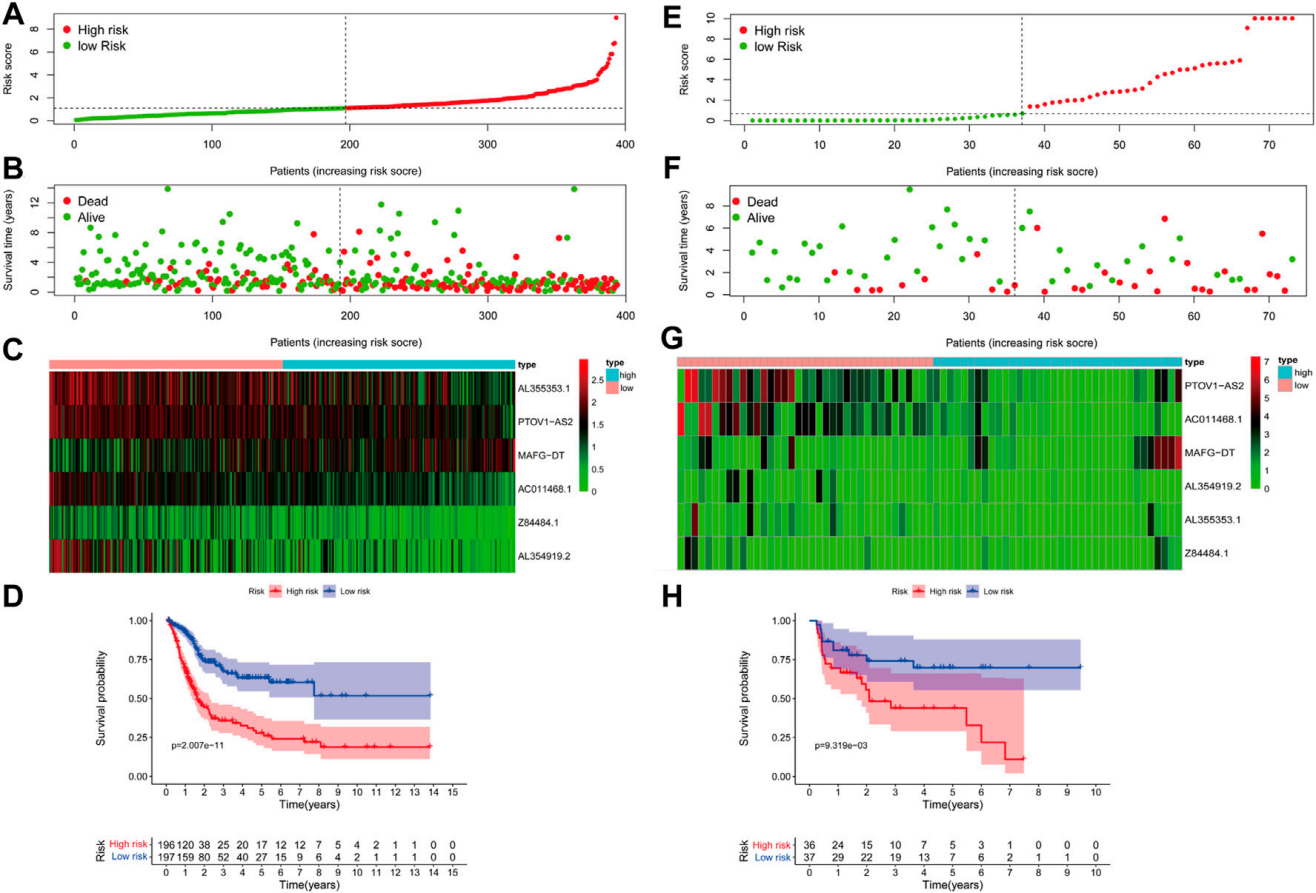
The accuracy of the prognostic model in training and testing cohort. **(A)** The distribution of risk score in TCGA BLCA cohort. **(B)** The survival status of high- and low-risk groups in TCGA BLCA cohort. **(C)** The expression of the 6 glyco-lncRNAs between the high- and low-risk groups in TCGA BLCA cohort. **(D)** Kaplan-Meier survival curve of the high- and low-risk groups in TCGA BLCA cohort. **(E–H)** The distribution of risk score, survival status, expression status of the 6 glyco-lncRNAs and Kaplan-Meier survival curve of the high- and low-risk groups in GSE154261 cohort.

In order to verify the availability, the BLCA cohort in TCGA database (training set) was subjected to the aforementioned model to get risk scores. Also, subsequently, patients were divided into low- and high-risk groups based on the median risk value (Figure 3A). By combining the risk score and the survival situation of patients, we could obviously conclude that the number of dead patients demonstrated a positive correlation with the value of risk score (Figure 3B). The expression of 6 glyco-lncRNAs in TCGA BLCA database was also analyzed. MAFG-DT was highly expressed, while AL355353.1, PTOV1-AS2, AC011468.1, and AL353919.2 were low expressed in the high-risk group (Figure 3C). The result of Kaplan–Meier (KM) survival curve indicated that the high-risk group demonstrated a shorter survival time when compared with low-risk group (Figure 3D). In addition, the BLCA cohort from GSE154261 (testing set) was also recruited to repeat the above analyses, aiming at further testing the effectiveness of our proposed model. The results of risk score, survival time, expression profile of the 6 glyco-lncRNAs, and Kaplan–Meier (KM) survival curve were roughly consistent with the results obtained from the BLCA cohort in TCGA database (Figures 3E–H). In summary, the high- and low-risk groups divided by our constructed model exhibit a discrepancy of survival time with statistical significance, indicating the high-efficiency of this prediction model to forecast the prognosis of BC patients.

### The Risk Score Can Predict Prognosis Independently and Tell the Difference Between Bladder Cancer Patients

**FIGURE 4:**
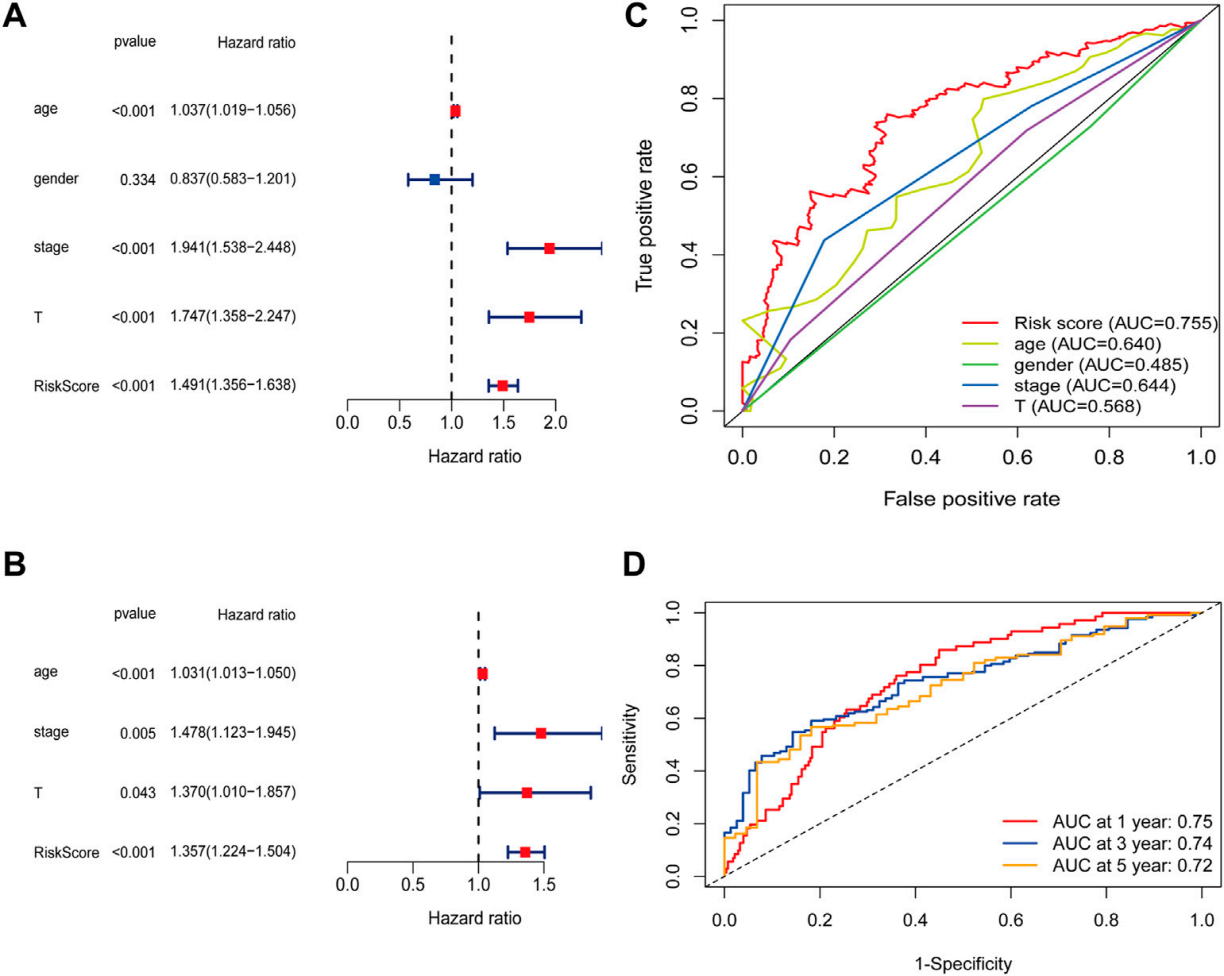
The risk score can predict prognosis independently. **(A)** Univariate COX analysis and **(B)** Multivariate COX analysis results of the risk score and common clinical characters. **(C)** The AUC of risk score and common clinical characters. **(D)** 1-, 3-, and 5-years ROC curves of the risk score.

The terms of gender, age, UICC stage, and T stage are common clinical prognosis indicators for BC patients (Tran et al., 2021). Making a comparison between the risk score obtained by our proposed model and the common indicators in the performance of predicting prognosis is essential. Both univariate and multivariate COX regression analyses demonstrated that the risk score could separately serve as an outstanding prognosis predictor for BC patients (Figures 4A, B). Furthermore, the result of time-dependent ROC curves showed that the 6-lncRNA prognostic model risk value was better than common clinical traits, with the AUC = 0.755 (Figure 4C). The results of 1-, 3-, and 5-year ROC curves were 0.75, 0.74, 0.72, respectively, which also showed an excellent prediction performance (Figure 4D).

**FIGURE 5:**
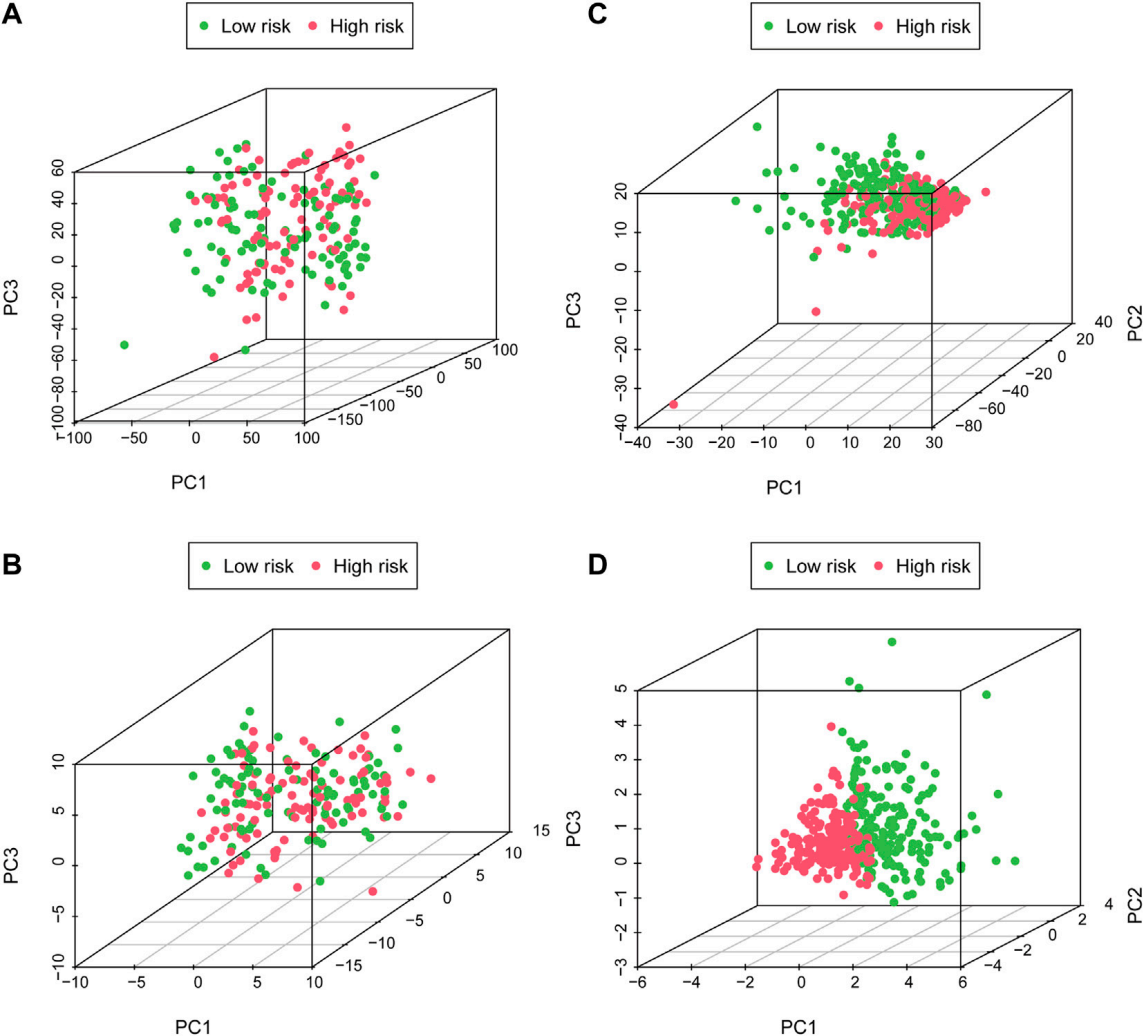
The high- and low-risk groups with different glycometabolism statuses. **(A–C)** PCA between the high- and low-risk groups on basis of whole gene sets, glycometabolism gene sets and glyco-lncRNAs. **(D)** PCA between the high- and low-risk groups according to the 6 glyco-lncRNAs.

PCA was performed to probe the distribution pattern discrepancy between high- and low-risk groups according to the whole gene set, glycometabolism-related gene, glyco-lncRNAs, and the 6 glyco-lncRNAs. The results verified that the 6 glyco-lncRNAs were able to separate high- and low-risk groups better than the others (Figures 5A–D). In other words, high-risk patients were quite distinguished from low-risk patients according to the 6 glyco-lncRNAs.

### High- and Low-Risk Groups Have Different Immune Cell Infiltration Status, Gene Set Enrichment Analysis Enrichment and Drug Sensitivity

**FIGURE 6:**
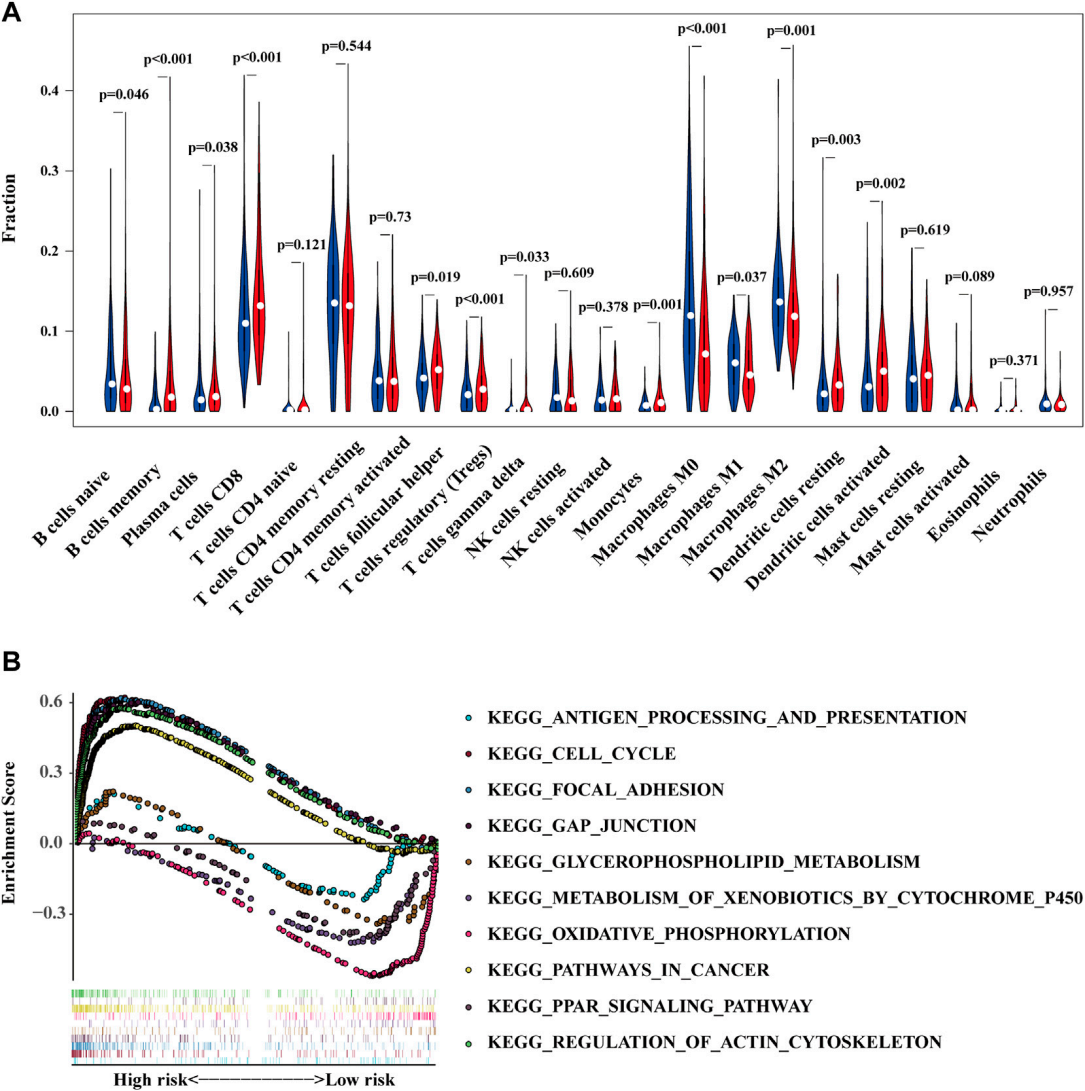
The high- and low-risk groups demonstrate different immune cell infiltration status **(A)** and enriched pathways of KEGG **(B)**.

The condition of immune cell infiltration in tumor microenvironment is one of the major effectors to decide the tumor development (Mbeunkui and Johann, 2009; Arneth, 2019). Therefore, the discrepancy of immune cell infiltration between the high- and low-risk groups was intended to be the next research content. As shown in Figure 6A, more B cells naïve, Macrophages M0, Macrophages M1, and Macrophages M2 were found in the high-risk group, while more B cells memory, plasma cells, T cells CD8, T cells follicular help, T cells regulatory, T cells gamma delta, Monocytes, Dendritic cells resting, and Dendritic cells were activated in the low-risk group. Afterwards, we also conducted a GSEA analysis to verify the different pathway enrichment of high- and low-risk groups. The results illustrated in Figure 6B revealed that the high-risk group enriched in pathways in cancer, cell cycle, gap junction, focal adhesion, and regulation of actin cytoskeleton. By contrast, the KEGG pathways of Antigen processing and presentation, Glycerophospholipid metabolism, Metabolism of xenobiotics by cytochrome P450, Oxidative phosphorylation, and PPAR signaling pathway were enriched in the low-risk group.

**FIGURE 7:**
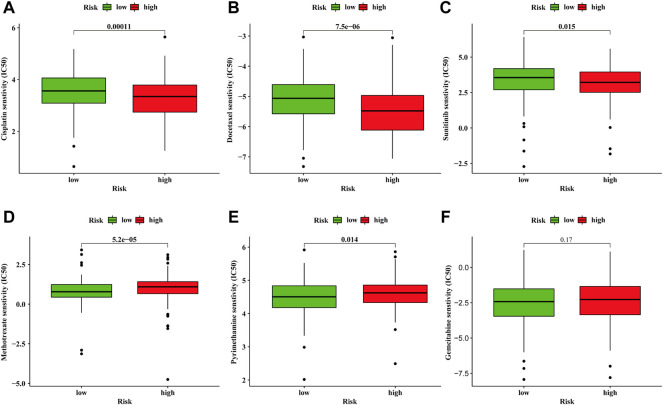
Discrepancy of drug sensitivity between high- and low-risk groups. The IC50 of cisplatin **(A)**, docetaxel **(B)**, sunitinib **(C)**, methotrexate **(D)**, pyrimethamine **(E)**, gemcitabine **(F)** between high- and low-risk groups.

Whether a tumor is sensitive to chemotherapy drugs is another determinant for the prognosis of BC patients (Kaufman et al., 2009; Lenis et al., 2020). We compared the sensitivity of common chemotherapeutic agents applied in clinical between high- and low-risk groups. The IC50 of Cisplatin generally used for BC treatment was lower in high-risk group (Figure 7A). Consistent with cisplatin, docetaxel, and sunitinib were also more sensitive in the high-risk group (Figures 7B, C). In regards to methotrexate and pyrimethamine, they might be more effective for the low-risk group (Figures 7D, E). However, the IC 50 of gemcitabine demonstrated no difference between high- and low-risk groups (Figure 7F). The above results indicate the proposed model exhibits a potential to facilitate the choice of a suitable treatment plan for BC patients.

### The Relation of 6 Glyco-lncRNAs With Clinical Traits

**FIGURE 8:**
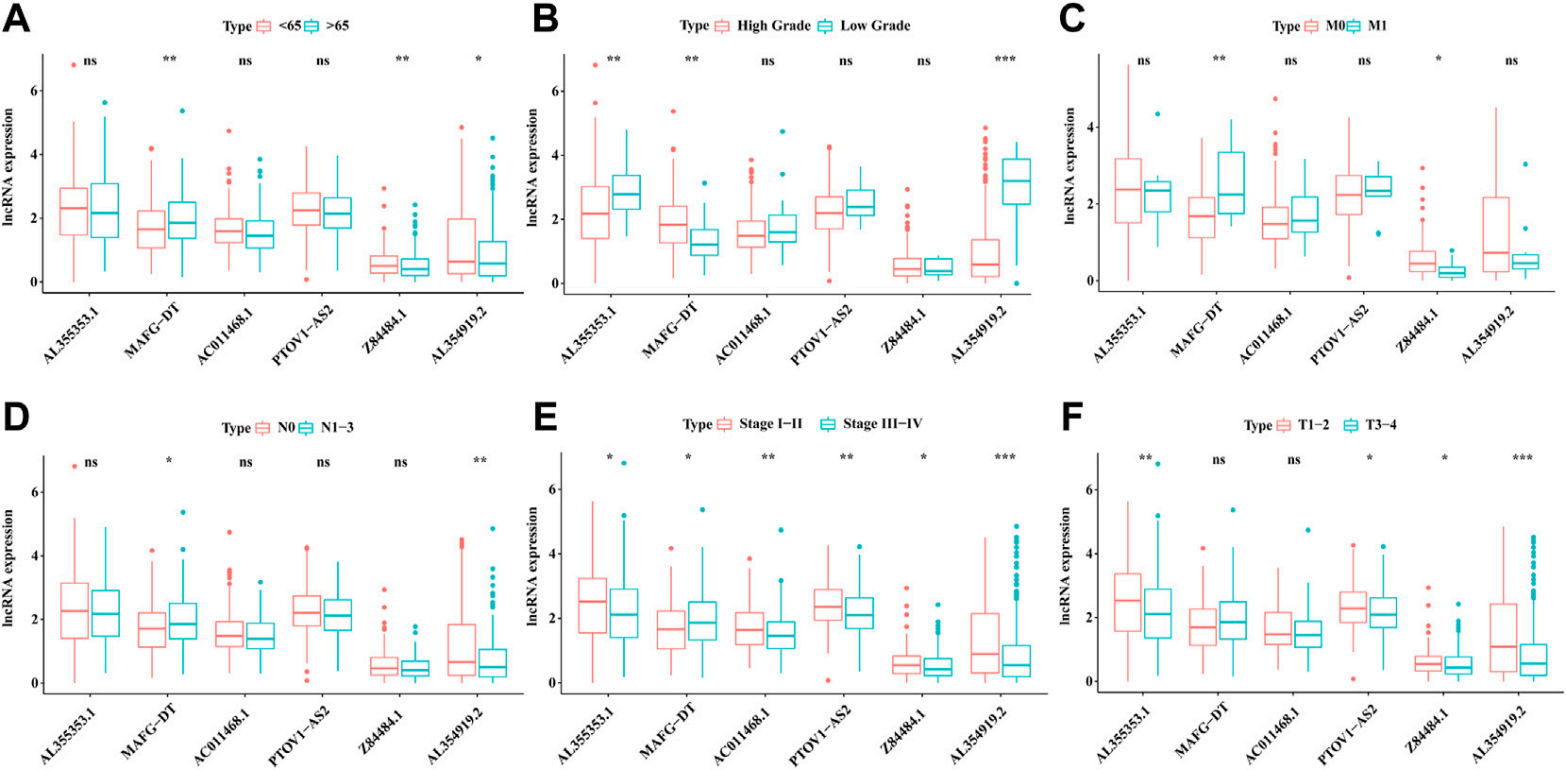
The association of 6 glyco-lncRNAs with clinical characters. The relation of the 6glyco-lncRNAs with age **(A)**, tumor grade **(B)**, distant metastasis status **(C)**, lymphatic metastasis status **(D)**, UICC stage **(E)**, and T stage **(F)**.

As mentioned above and shown in Table 1, the prediction prognosis model for BC patients totally included 6 glyco-lncRNAs (AL355353.1, MAFG-DT, AC011468.1, PTOV1-AS2, Z84484.1, and AL354919.2). Then, we intended to reveal the relation between the expression of these 6 glyco-lncRNAs and clinical characters such as age, grade, metastatic status, UICC stage, and T stage. In aspect of age, MAFG-DT showed a higher expression level in BC patients older than 65 years, while the expression of Z84484.1 and AL354919.2 was repressed in older patients (Figure 8A). Besides, we also found that the expression of AL355353.1 and AL354919.2 was significantly upregulated in low-grade patients, while MAFG-DT was downregulated (Figure 8B). For the expression relation with the stage, MAFG-DT demonstrated an increased tendency in M1 and N1-3 patients, while Z84484.1 and AL354919.2 demonstrated a decreased tendency in M1 patients and N1-3 patients, respectively (Figures 8C, D). All these 6 glyco-lncRNAs demonstrated a positive correlation with UICC stage, and AL354919.2 was obviously suppressed in Stage Ⅲ–Ⅳ and T3-4 patients (Figures 8E, F).

### Expression of the 6 Glyco-lncRNA in Tissues and Urine Exosomes of Bladder Cancer Patients

**FIGURE 9:**
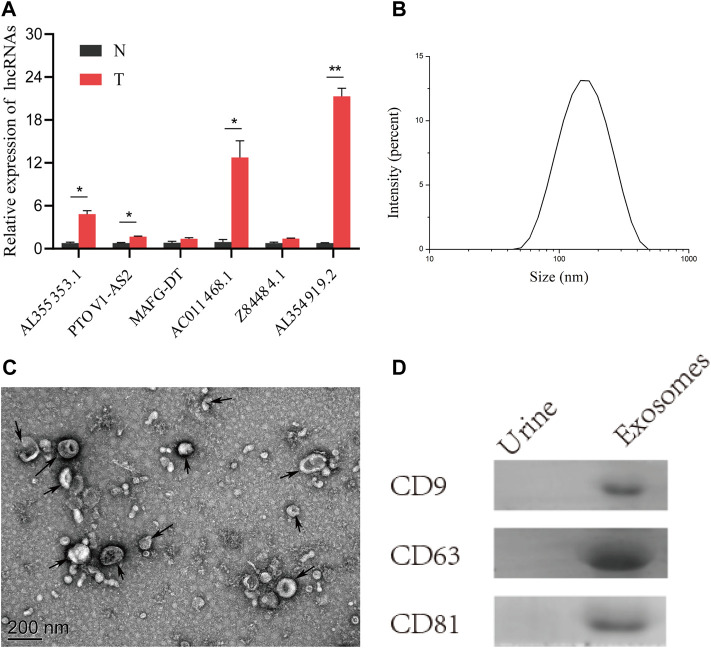
Tumor tissues had an obvious high-expression of AL355353.1, AC011468.1, and AL354919.2. **(A)** The expression of the 6 glyco-lncRNAs in BC tissues and paired normal tissues. **(B)** Measurement of particle size of the urine exosomes. **(C)** Representative electron microscopy image of the urine exosomes from BC patients. **(D)** Western blot analysis of the exosomal protein markers CD9, CD63, and CD81. *p < 0.05, **p < 0.01.

To verify the practicality of the 6 glyco-lncRNAs prognostic model, we detected their expressions in BC tissues and paired normal paracancerous tissues, as well as in urine exosomes of BC patients with RT-qPCR method. The result confirmed that all of these 6 glyco-lncRNAs demonstrated an increased expression level in BC tissues when comparing with normal paracancerous tissues, and three of them (AL355353.1, AC011468.1, and AL354919.2) were attached with statistically significance (Figure 9A). LncRNAs containing in exosomes have been recognized as biomarkers for the diagnosis and prognosis of various cancers (Naderi-Meshkin et al., 2019). The contents detection of urine exosomes as a noninvasive method exhibits the ability to diagnose diseases of the urinary system including BC (Yazarlou et al., 2018). Therefore, we wondered whether these 6 glyco-lncRNAs exist in the exosomes of BC patients’ urine and can serve as biomarkers. We collected urine samples of BC patients and then isolated exosomes. The characterization assays including particle size detected by Zetasizer Lab, morphology feature, and exosomal protein markers demonstrated we purified exosomes successfully (Figures 9B–D). It was very unexpected that the amplification instrument could not give valid results for all of these 6 glyco-lncRNAs, except the internal reference gene (Supplementary Table S2), indicating that there were less of them in urine exosomes to be detected by RT-qPCR.

## Discussion

Due to the quite high recurrence rate of BC, patients have to suffer regular cystoscopy and be followed up in a long term, resulting in a high treatment cost. Nonetheless, with the further tumor progression, radical cystectomy will be considered as the best therapy method in clinical, which would extremely bring down the living quality of BC patients. Therefore, great attention should be paid to early prognosis prediction. Next, the Warburg Effect indicates that cancer cells own a special hallmark of energy metabolism (Faubert et al., 2020). LncRNAs have been verified to involve in the metabolic reprogramming of cancer cells via impacting the expression of metabolism-associated genes and can be functioned as prognostic indicators for cancers (Boque-Sastre and Guil, 2020). However, an effective prognostic model for BC patients based on glyco-lncRNAs is lacking so far.

Previous studies verified that lncRNAs take part in the progression of cancer by acting as regulators and can predict the prognosis of BC patients (Huarte, 2015; Esposito et al., 2019). A signature including five lncRNAs reported by Zhang et al. (2020) can predict the prognosis of bladder urothelial carcinoma. Also, they also preliminarily explored the underlying mechanism. Wang et al. (2021) constructed a prognosis prediction model including seven immune-related lncRNAs for BC. However, these signatures were not tested in a validating cohort or detected in clinical samples. Besides, the AUC value in our study was 0.755, which was superior to existing studies based on Immune-related lncRNA, Extracellular matrix-related lncRNA, and Autophagy-related lncRNA, whose AUC value were 0.642, 0.686, and 0.71, respectively (Qing et al., 2020; Sun et al., 2020; Luo et al., 2021). The prediction model we constructed here contained 6 glyco-lncRNAs, including AL355353.1, AC011468.1, AL354919.2, Z84484.1, PTOV1-AS2, and MAFG-DT. Compared with normal paracancerous tissues, tumor tissues had an obvious high-expression of AL355353.1, AC011468.1 and AL354919.2. Among them, AL355353.1 was upregulated in BC patients with a low-grade, while being downregulated in T3-4 and Stage Ⅲ–Ⅳ patients, indicating that AL355353.1 demonstrates a negative association with BC progression. However, no studies exist on its biological function in BC. Mechanism exploration of AL355353.1 in BC may invoke great interest for researchers and demonstrate profound significance. The expression of another prognosis-related lncRNA AC011468.1 was repressed in Stage Ⅲ–Ⅳ, which is consistent with the report by Zhao et al. (2021). Also, AL354919.2, Z84484.1, PTOV1-AS2, and MAFG-DT were found to be associated with the prognosis of cancer patients in several studies (Liu et al., 2021; Su et al., 2021; Wang et al., 2021; Zhao et al., 2021). Also, especially, AL354919.2 was obviously upregulated in low-grade BC patients. Consistent with the common biological phenomena, AL354919.2 was downregulated in T3-4 patients, Stage Ⅲ–Ⅳ patients, and patients with lymph node metastasis, which meant that AL354919.2 may restrain the progression of BC.

Exosomes are extracellular vesicles with a diameter of 50–150 nm secreted by living cells and exist in a variety of body fluids, such as urine, prostate fluid, serum, breast milk, and so on (Ruivo et al., 2017). As the main vehicles for cell communication, exosomes are loaded with many functional proteins, DNA, messenger RNA, and some non-coding RNA by their parent cells (Wang et al., 2020). LncARSR transferred by exosomes derived from Sunitinib resistance renal cells confers resistance to sensitive cells (Qu et al., 2016). In this study, the 6 glyco-lncRNAs used to construct the prediction model were not detected in urinary exosomes. The major reason we speculated is that there are too less of these glyco-lncRNAs being loaded due to the precise sorting mechanisms (Villarroya-Beltri et al., 2013). In addition, exosomes are proved to contain a large amount of short RNAs (< 200 bp) such as microRNAs, which might ascribe from their limited volume (Shurtleff et al., 2017; Han et al., 2020). To our knowledge, we explored the relations between these 6 glyco-lncRNAs and clinical traits, and we detected them in tissue and urine exosomes samples for the first time.

TNM staging system is still a standard method for clinicians and medical scientists to assess the disease state of BC patients. However, the TNM staging system mainly confines to the anatomical level, and it is underrepresented in the prediction of cancer biology (Qu et al., 2018). The pathological grading system for BC depends on the subjective judgment of pathologists. The prediction model for the prognosis of BC we constructed is capable of complementing these deficiencies. Next, the risk score suggested by the prediction model could predict the prognosis of patients with BC both in TCGA cohort and GSE154261 cohort accurately. Compared with other clinical indicators, including age, sex, AJCC stage and T stage, the risk value was a forceful independent prognosis factor for BC patients. High- and low-risk groups were obviously distributed in two different directions based on the 6 glycol-lncRNAs, indicating the efficacy of this lncRNA signature. As a consequence, the glyco-lncRNA signature demonstrated a promising prospect for clinical application and laid the foundation for further research of glycometabolism alteration.

Immune cells infiltration in tumor microenvironment (TME) plays an important role in tumor progression, immune escape, and drug resistance (Turley et al., 2015). Our results showed that B cells naïve, Macrophages M0, M1, and M2 aggregated more in the high-risk group. Also, macrophages usually promote tumor progression and high infiltration of them is related to poor prognosis of cancer patients (Ryder et al., 2008; Zhou et al., 2009). Targeting therapy to the macrophages in TME can inhibit the proliferation of tumor (Hiroshima et al., 2014). GSEA results showed that pathways in cancer, cell cycle, gap junction, focal adhesion, and regulation of actin cytoskeleton were mainly enriched in the high-risk group. Deficiency in regulating the cell cycle causes the sustained proliferation of cancer cells, which is a hallmark of cancer development. For example, heterodimeric protein complexes holding a kinase activity are major cell cycle regulators. WEE1, which can inhibit the function of CDK1-cyclinB complex, is a potential target for cancer therapy (Vakili-Samiani et al., 2022). Also, ZNF703 can induce G1-phase arrest and is an important protein involved in triple-negative breast cancer progression (Zhang et al., 2022).

In the present study, we constructed a prognosis prediction model based on glyco-lncRNAs for BC patients, and its efficiency was validated both in training cohort and testing cohort. The model of glyco-lncRNAs demonstrates a better performance in predicting OS of BC patients than the other clinical characters. Meanwhile, the prognostic model is able to significantly separate BC patients into two groups with different immune cell infiltration conditions, enriched pathways, and chemotherapy sensitivity. In addition, we found that glyco-lncRNAs AL355353.1, AC011468.1, and AL354919.2 demonstrated significantly high expressions in tumor tissues.

## Data Availability

The original contributions presented in the study are included in the article/Supplementary Material, and further inquiries can be directed to the corresponding authors.
