# Supplementary material for: Recognition of Glycometabolism-Associated lncRNAs as Prognosis Markers for Bladder Cancer by an Innovative Prediction Model
Source: Front Genet. 2022 Jul 19;13:918705. doi: 10.3389/fgene.2022.918705 (PMC9343799; doi:10.3389/fgene.2022.918705)
Supplement: Supplementary file 1 [file Table1.docx]

**Supplementary Table 1.** Primer sequences used to amplify the target lncRNAs by RT-qPCR.

| gene name |  | Primer sequence (5’-3’) |
| --- | --- | --- |
| GAPDH | Forward | CATCATCCCTGCCTCTACTG |
|  | Reverse | GCCTGCTTCACCACCTTC |
| AL355353.1 | Forward | AGGCTCGGAACAGCTT |
|  | Reverse | TCCTCGAGGCTGCTTCG |
| MAFG-DT | Forward | ACACTGGCTGCTCTAGC |
|  | Reverse | AGGCGAGGCAGGCTC |
| AC011468.1 | Forward | ATGGTGCTGGGATTACAG |
|  | Reverse | TGTTCCCTTGTACTTCAGTG |
| Z84884.1 | Forward | GAAGGGGCTCAAGGCACAG |
|  | Reverse | AGTTCAGGTGTTGCTGCATT |
| PTOV1-AS2 | Forward | AGTCAGCTGAGCCTCC |
|  | Reverse | CCTTGTCACTCCTGGTTT |
| AL354919.2 | Forward | AACTCCTTGCGCTAAGTTCC |
|  | Reverse | CCTGCTGGACTGGAAG |
