# Supplementary material for: Recognition of Glycometabolism-Associated lncRNAs as Prognosis Markers for Bladder Cancer by an Innovative Prediction Model
Source: Front Genet. 2022 Jul 19;13:918705. doi: 10.3389/fgene.2022.918705 (PMC9343799; doi:10.3389/fgene.2022.918705)
Supplement: Supplementary file 2 [file Table2.docx]

**Supplementary Table 2.** The CT values of target genes detecting by RT-qPCR

| gene name |  | CT value（Mean±SD） |
| --- | --- | --- |
| GAPDH |  | 36.067±0.658 |
| AL355353.1 |  | Undetermined |
| MAFG-DT |  | Undetermined |
| AC011468.1 |  | Undetermined |
| Z84884.1 |  | Undetermined |
| PTOV1-AS2 |  | Undetermined |
| AL354919.2 |  | Undetermined |
